# A farewell to outgoing Editor-in-Chief Michel Brémont

**DOI:** 10.1186/s13567-019-0711-6

**Published:** 2019-11-11

**Authors:** Elodie Coulamy, Vincent Béringue

**Affiliations:** Veterinary Research, UR892 VIM, INRA, Jouy-en-Josas, France

Dr Michel Brémont has served as Editor-in-Chief since 2009. Michel retired at the beginning of May 2019, after a life dedicated to science. Michel was a scientist specialized in virology, working in the Virology and Immunology Unit at the Animal Health Division of the French National Institute for Agricultural Research (INRA). First focused on rotavirus, he then became a leader on fish viruses and the development of viral vaccines. He has authored more than seventy research articles or reviews on these topics.

*Veterinary Research* is a peer-reviewed journal that publishes high quality and novel research and review articles that contribute significantly to all aspect of infectious diseases and host–pathogen interactions. *Veterinary Research* has been edited by INRA since 1993. It is supported by INRA Animal Health Division. Before, it was published under the title *Annales de Recherches Vétérinaires*, which was created in 1970.

Michel has been instrumental in the transformation of *Veterinary Research* as an open access journal published with BMC since 2011. As Editor-in-Chief, he created a board of associate editors. He was himself in charge of the Virology section. With their contribution, he made of *Veterinary Research* a high-quality journal with a high impact. *Veterinary Research* publishes original articles and reviews on infectious diseases from around the globe. Each year, our editors receive more than 350 manuscripts, approximately a-third of which are published.

We would like to take the opportunity of this editorial to recall that *Veterinary Research* provides the highest possible level of editorial service. Our associate editors and Editor-in-Chief  are dedicated for communicating scientifically sound work to the largest possible audience. Our editors cover all the field of infectiology and the host–pathogen interactions, including immunology and epidemiology. All pathogens are considered including bacteria, viruses, parasites and prions. Studies on zoonotic and emerging infections and on adaptation to climate change or consequences of climate change are highly appreciated.

Farewell Michel, and enjoy life in the South-West of France.

